# Supplementary material for: Tristetraprolin binding site atlas in the macrophage transcriptome reveals a switch for inflammation resolution
Source: Mol Syst Biol. 2016 May 13;12(5):868. doi: 10.15252/msb.20156628 (PMC4988506; doi:10.15252/msb.20156628)
Supplement: Supplementary file 1 — Expanded View Figures PDF [file MSB-12-868-s001.pdf]

Expanded View Figures

Figure EV1. Quality control for PAR-iCLIP experiment.

A Autoradiograph image showing TTP–RNA complexes on denaturing bis-tris 4–12% gradient gel after membrane transfer. Membrane was exposed to phosphorimager screen for 1 h. RNA was labeled by 3′ end ligation of ^{32}P-RNA linker. Increasing concentration of RNase I (as indicated lanes 1–4) results in decreasing size of the TTP–RNA complexes. Lanes 5 and 6 contain biological replicates with intermediate RNase I concentration. No signal was detected in samples from ΔM BMDMs (lanes 7–9).

B Nucleotide composition at and around positions 0 of PAR-iCLIP reads. Position 0 is defined as position 1 nt upstream of the 5′ end of reads. Note the increased frequency of U at position 0 resulting from crosslink-driven termination of reverse transcription.

C Frequency of single nucleotide substitutions in PAR-iCLIP reads.

D Overlap between position 0 of reads (i.e. reverse transcription termination) and TC transitions in reads spanning the same region; 48% of all positions 0 overlap with at least 1 TC transition.

E Same as (D), but only crosslink positions inside the TTP binding sites were selected for analysis of overlap with TC transitions; 80% of all positions 0 in the TTP binding sites overlap with at least 1 TC transition.

F Similar to (B), but RNA-Seq reads were analyzed instead of PAR-iCLIP reads. Note that all nucleotides occur at equal frequency at and around the position 0.

G Same as (C), but RNA-Seq reads were analyzed instead of PAR-iCLIP reads. Note that all substitutions occur at similar frequency.

Source data are available online for this figure.

 © 2016 The Authors

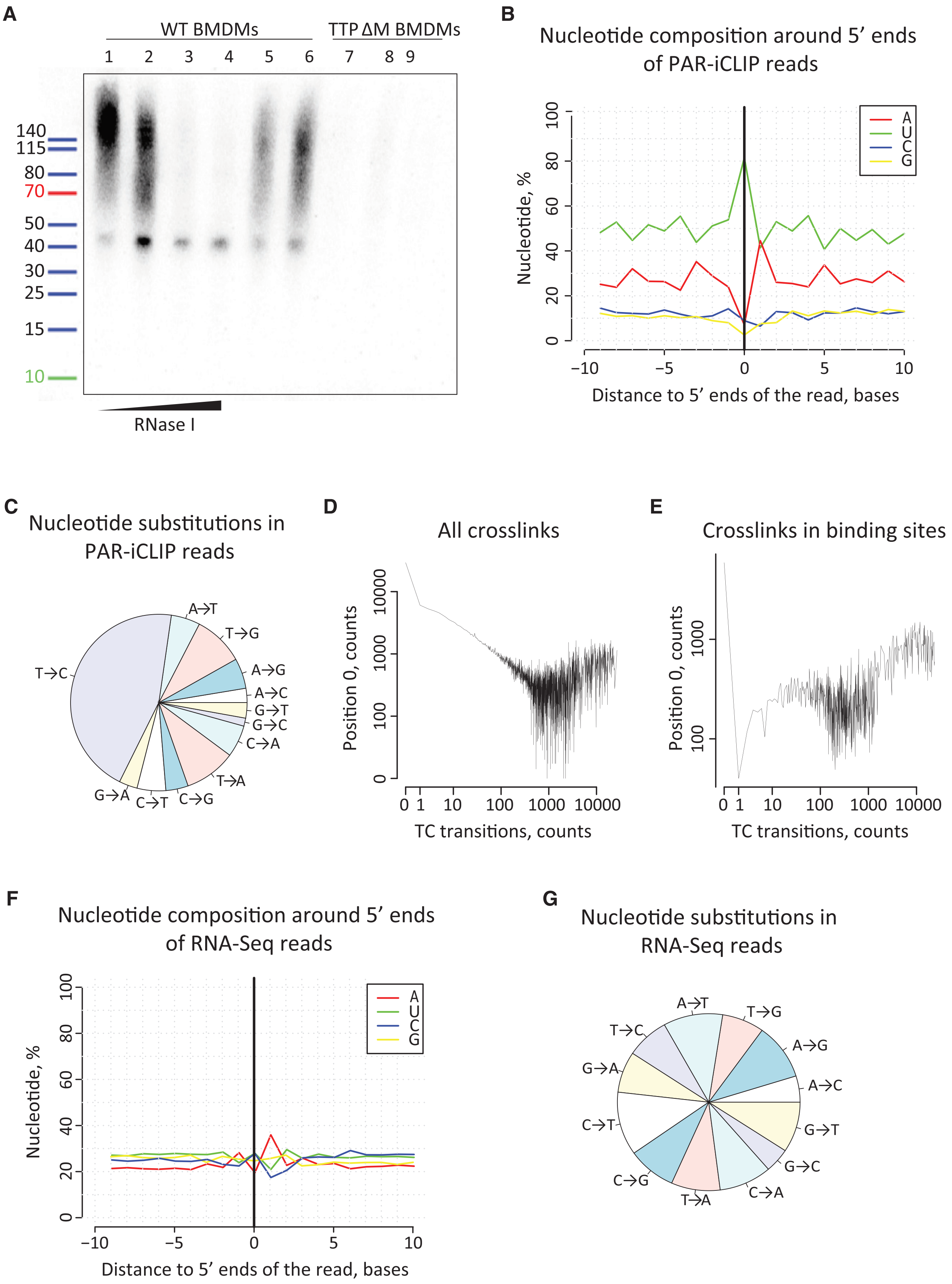
A
WT BMDMs
TTP ΔM BMDMs
1 2 3 4 5 6 7 8 9
140
115
80
70
50
40
30
25
15
10
RNase I
B
Nucleotide composition around 5’ ends of PAR-iCLIP reads
Nucleotide, %
Distance to 5’ ends of the read, bases
A
U
C
G
C
Nucleotide substitutions in PAR-iCLIP reads
A→T
T→G
A→G
A→C
G→T
G→C
C→A
T→A
C→G
C→T
G→A
T→C
D
All crosslinks
Position 0, counts
TC transitions, counts
E
Crosslinks in binding sites
Position 0, counts
TC transitions, counts
F
Nucleotide composition around 5’ ends of RNA-Seq reads
Nucleotide, %
Distance to 5’ ends of the read, bases
G
Nucleotide substitutions in RNA-Seq reads

Figure EV1.

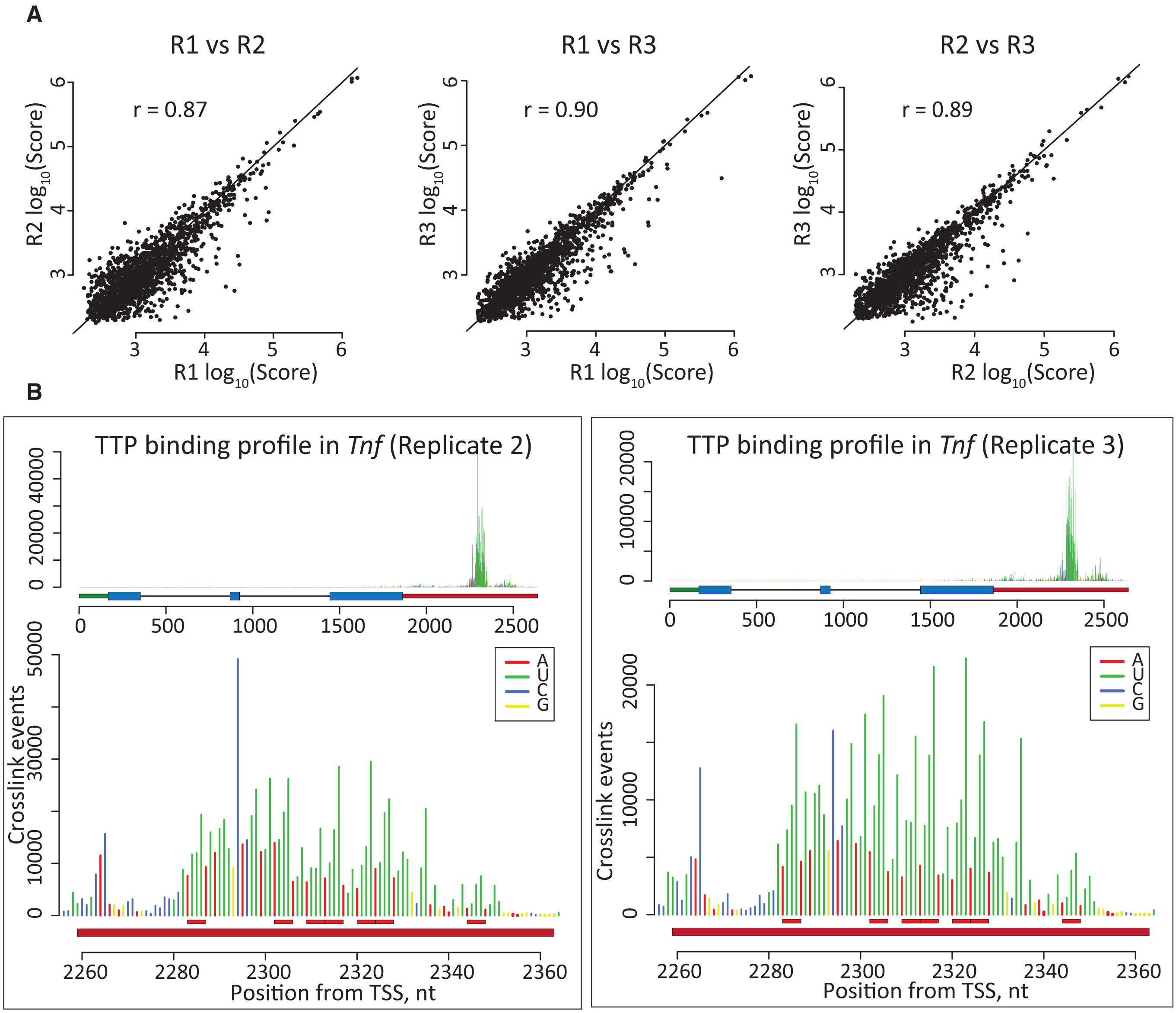

Figure EV2. Comparison of PAR-iCLIP replicates.

A Scatter plots comparing log-transformed scores of TTP binding sites in three biological replicates (R1–R3). TTP binding sites were identified in each replicate using Pyicos. Pearson's correlation coefficients for TTP binding sites among the replicates were ranging from 0.87 to 0.90.

B TTP binding profile on the *Tnf* transcript (GenBank accession no. NM_013693) in biological replicates R2 and R3. The profile in replicate R1 is shown in Fig 1E.

© 2016 The Authors

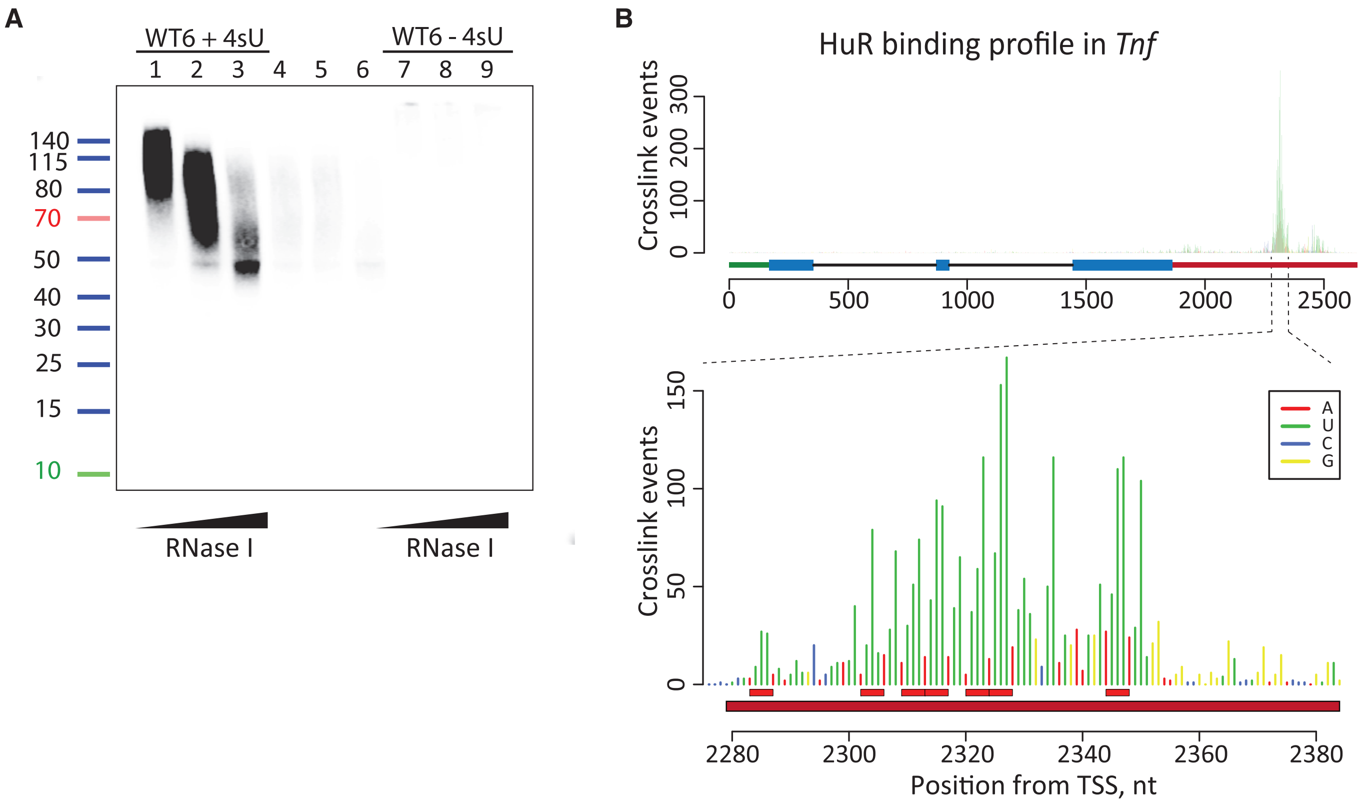

Figure EV3. HuR PAR-iCLIP.

A Autoradiograph image showing HuR–RNA complexes on denaturing bis-tris 4–12% gradient gel after membrane transfer. Membrane was exposed to phosphorimager screen for 1 h. RNA was labeled by 3′ end ligation of ^{32}P-RNA linker. Increasing concentration of RNase I (as indicated) results in a decreasing size of the HuR–RNA complexes (lanes 1–3). No signal is detectable when crosslinking was performed without prior addition of 4-thiouridine (lanes 7–9).

B HuR binding profile on *Tnf* transcript.

Source data are available online for this figure.

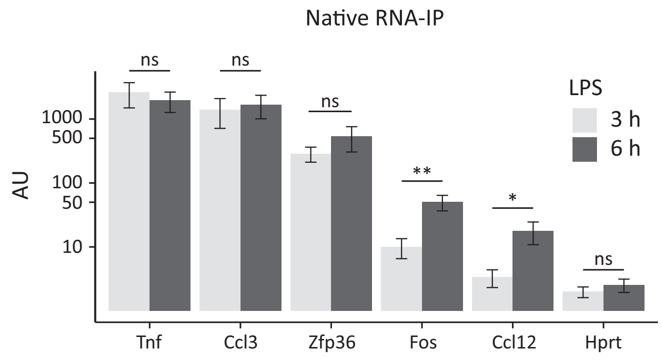

Figure EV4. Native TTP–RNA-IP.

Amount of mRNA co-precipitated with TTP increases for *Fos* and *Ccl12*, with time of LPS treatment (3 and 6 h), but remains unchanged for *Tnf*, *Ccl3*, and *Zfp36*. AU, arbitrary units. Difference between group means was tested with two-sample unequal variance *t*-test. Error bars represent 95% confidence interval, $n = 3$ biological replicates. $^{*}P < 0.05$; $^{**}P < 0.01$; ns, not significant.

Figure EV5. Decay rates of *Cxcl1*, *Ccl4*, *Il10*, *Zfp36l2*, and *Irf1* mRNAs assessed by qRT–PCR.

WT and ΔM BMDMs were stimulated for 3 h (left panels) or 6 h (middle panels) with LPS and transcription was stopped by actinomycin D (ActD) followed by measurements of remaining mRNA 45 and 90 min after the transcription blockage. Stability parameters as well as statistical testing of differences in slopes were calculated using linear model. Shaded areas around regression lines represent 95% confidence intervals for linear regression, $n = 3$ biological replicates; *P*-values are indicated in the graphs.

▶

© 2016 The Authors

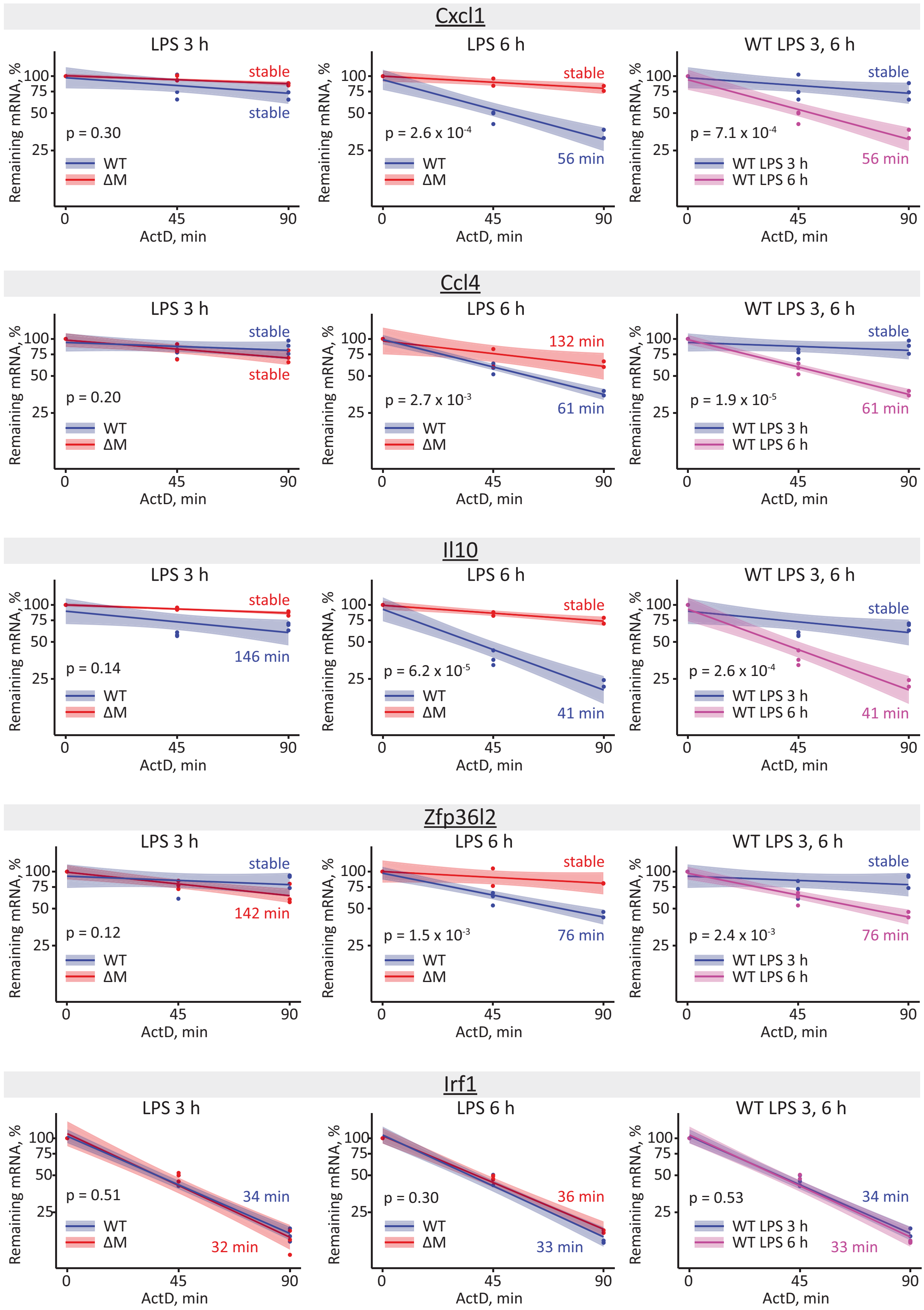

Figure EV5.

© 2016 The Authors
