# Supplementary figures and images for: Tristetraprolin binding site atlas in the macrophage transcriptome reveals a switch for inflammation resolution
Source: Mol Syst Biol. 2016 May 13;12(5):868. doi: 10.15252/msb.20156628 (PMC4988506; doi:10.15252/msb.20156628)

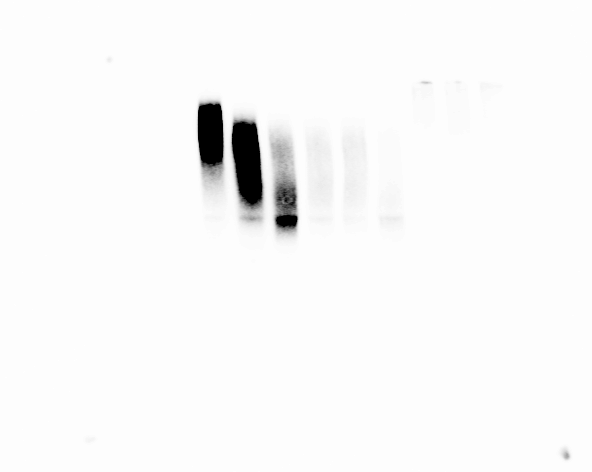

Supplement: Supplementary file 15 — Source Data for Expanded View [file MSB-12-868-s014.zip › Source_Data_EV_Figures/EV3A.tif]

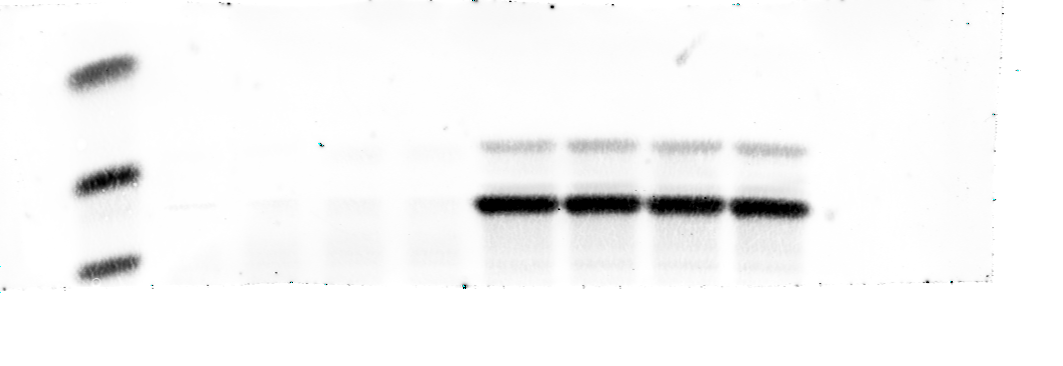

Supplement: Supplementary file 16 — Source Data for Figure 3G [file MSB-12-868-s015.zip › Source_Data_Figure_3G/3G_H3.tif]

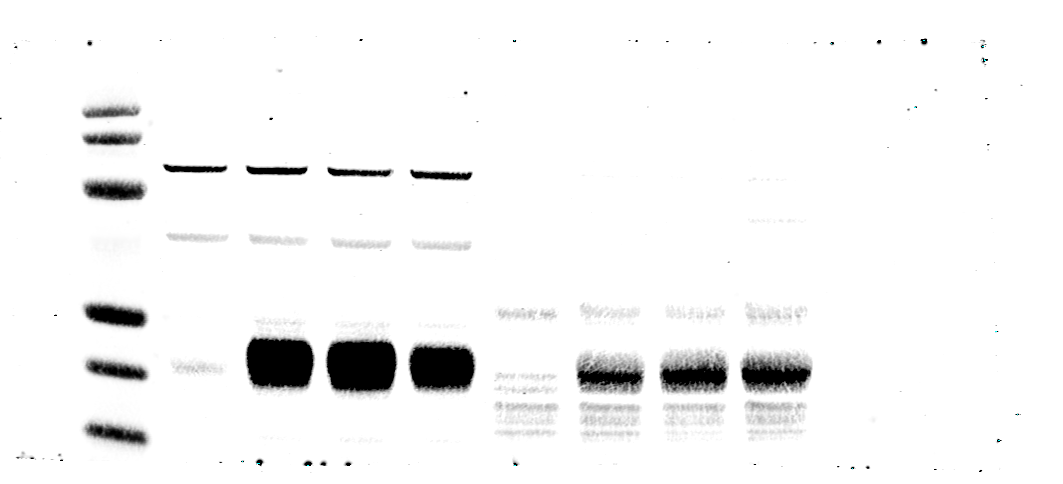

Supplement: Supplementary file 16 — Source Data for Figure 3G [file MSB-12-868-s015.zip › Source_Data_Figure_3G/3G_TTP.tif]

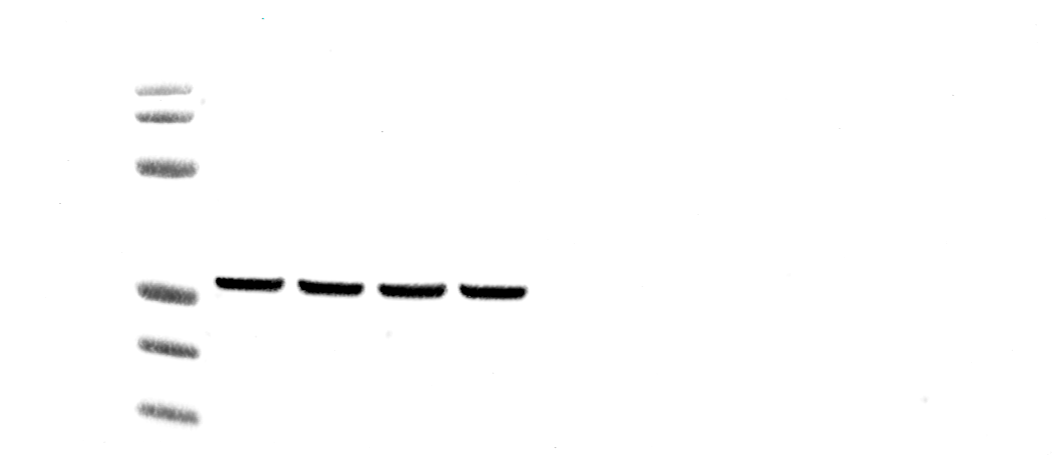

Supplement: Supplementary file 16 — Source Data for Figure 3G [file MSB-12-868-s015.zip › Source_Data_Figure_3G/3G_Tub.tif]

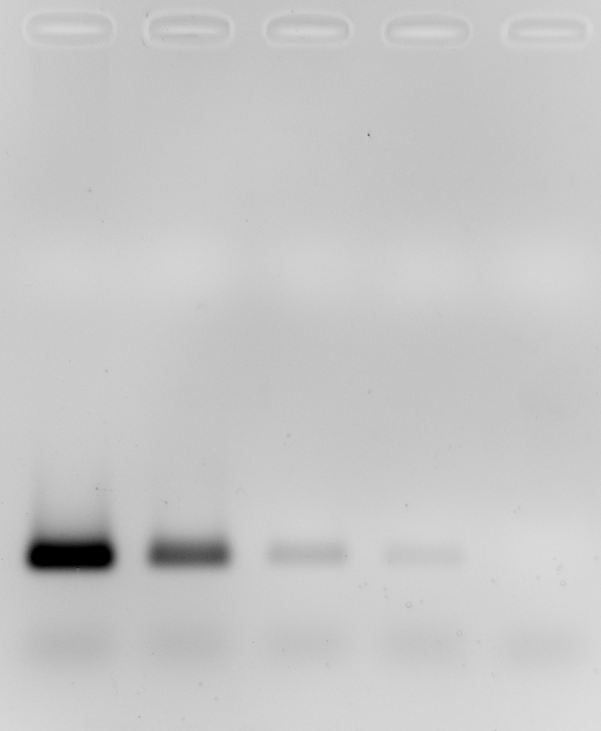

Supplement: Supplementary file 17 — Source Data for Figure 4H [file MSB-12-868-s016.zip › Source_Data_Figure_4H/4H_CxcL2.tif]

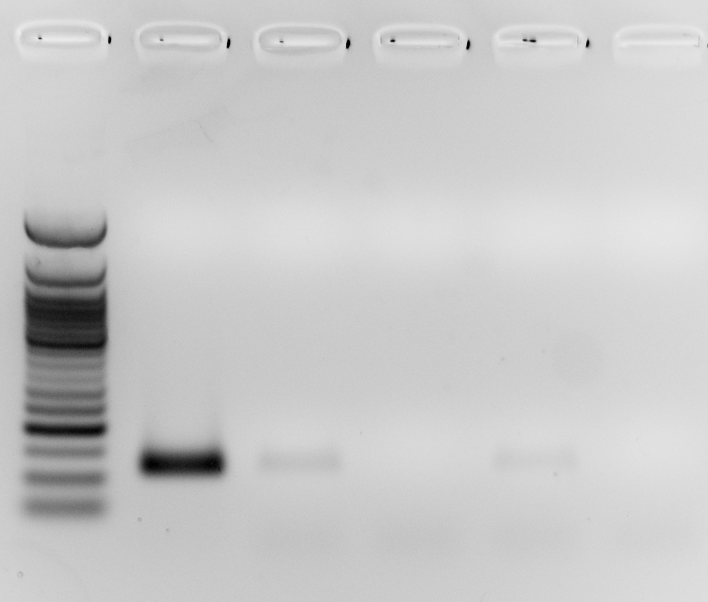

Supplement: Supplementary file 17 — Source Data for Figure 4H [file MSB-12-868-s016.zip › Source_Data_Figure_4H/4H_Gnb1.tif]

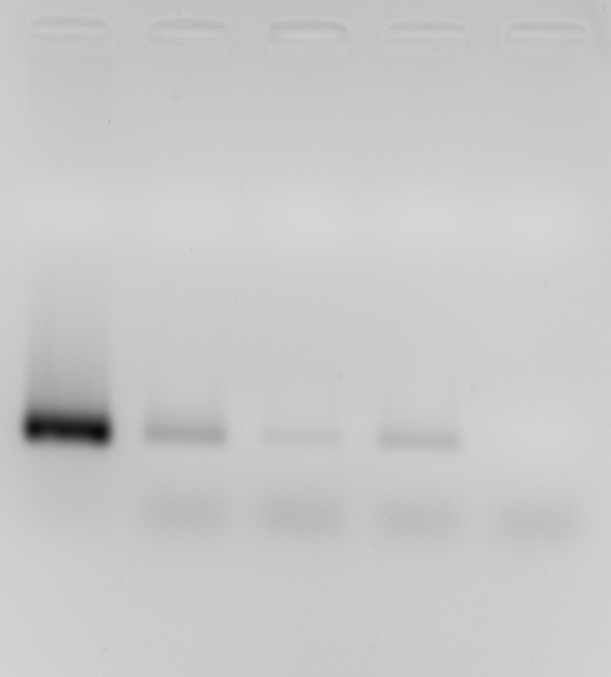

Supplement: Supplementary file 17 — Source Data for Figure 4H [file MSB-12-868-s016.zip › Source_Data_Figure_4H/4H_Hprt.tif]

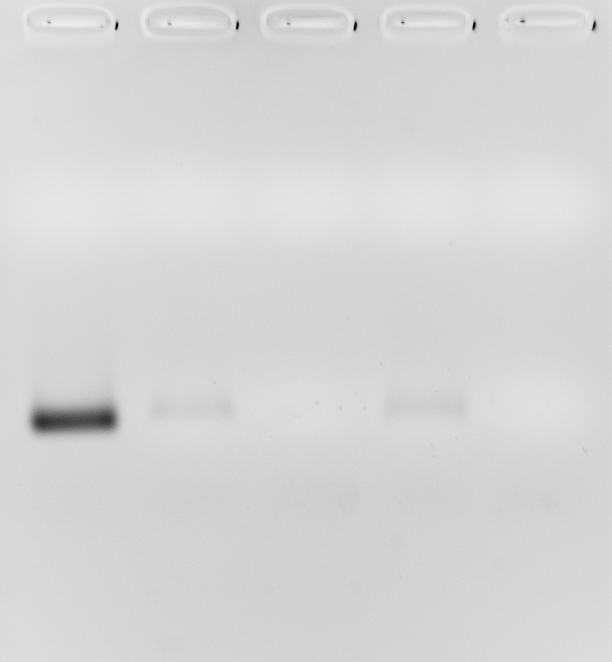

Supplement: Supplementary file 17 — Source Data for Figure 4H [file MSB-12-868-s016.zip › Source_Data_Figure_4H/4H_Tapbp.tif]

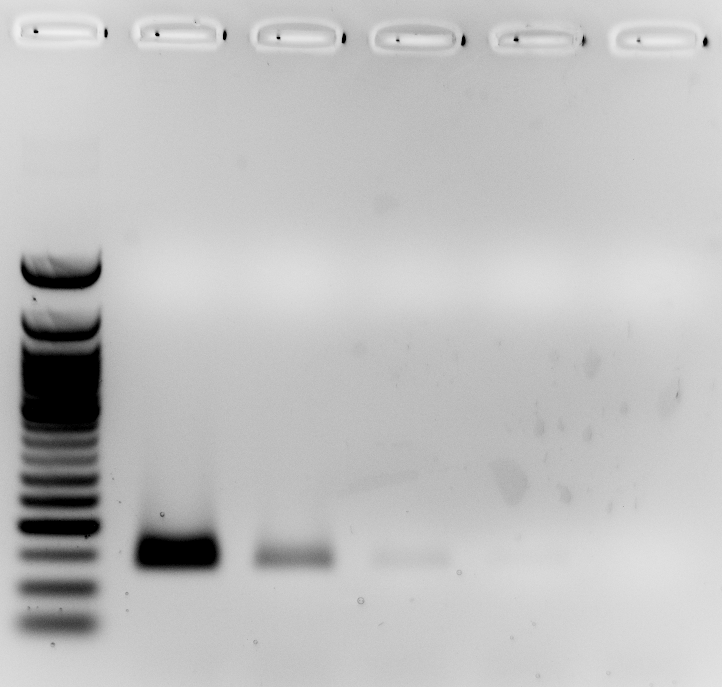

Supplement: Supplementary file 17 — Source Data for Figure 4H [file MSB-12-868-s016.zip › Source_Data_Figure_4H/4H_Tnf.tif]
